# Detection and characterisation of optic nerve and retinal changes in primary congenital glaucoma using hand-held optical coherence tomography

**DOI:** 10.1136/bmjophth-2018-000194

**Published:** 2019-06-24

**Authors:** Anastasia V Pilat, Sonal Shah, Viral Sheth, Ravi Purohit, Frank A Proudlock, Joseph Abbott, Irene Gottlob

**Affiliations:** 1 Ophthalmology Group, University of Leicester, Leicester, UK; 2 Ophthalmology, University of Leicester, Leicester, UK; 3 Ophthalmology, Birmingham Children’s Hospital, Birmingham, UK

**Keywords:** child health (paediatrics), diagnostic tests/investigation, imaging

## Abstract

**Objective:**

To investigate (1) the feasibility of scanning the optic nerve (ON) and central retina with hand-held optical coherence tomography (HH-OCT) without sedation or anaesthesia in primary congenital glaucoma (PCG), (2) the characteristics of ON changes in comparison with adult primary open-angle glaucoma (POAG) in comparison with matched controls, (3) the sensitivity and specificity of ON parameters for diagnosis, and (4) changes of foveal morphology.

**Methods and analysis:**

HH-OCT (Envisu 2300; Leica Microsystems) was used to investigate ON and foveal morphology of 20 children with PCG (mean age 4.64±2.79) and 10 adult patients with POAG (mean age 66.8±6.94), and compared with age-matched, gender-matched and ethnicity-matched healthy controls without sedation or anaesthesia.

**Results:**

HH-OCT yielded useful data in 20 out of 24 young children with PCG. Patients with PCG had significantly deeper cup changes than patients with POAG (vs respective age-matched controls, p=0.014). ON changes in PCG are characterised by significant increase in cup depth (165%), increased cup diameter (159%) and reduction in rim area (36.4%) as compared with controls with high sensitivity (81.5, 74.1% and 88.9%, respectively) and specificity (85.0, 80.0% and 75.0%, respectively). Patients with PCG have a significantly smaller width of the macula pit (p<0.001) with non-detectable external limiting membrane.

**Conclusion:**

HH-OCT has the potential to be a useful tool in glaucoma management for young children. We have demonstrated the use of HH-OCT in confirming a diagnosis of glaucoma within the studied cohort and found changes in disc morphology which characterise differently in PCG from POAG.

**Key messages**

**What is already known about this subject?**

- Optical coherence tomography (OCT) is widely used in older patients with glaucoma in whom normative ranges have been established and patterns of disease are well described.

**What are the new findings?**

- Using hand-held OCT (HH-OCT) in a younger group of patients (mean age=4.64 years), our novel findings in primary congenital glaucoma were a particularly increased cup depth (vs primary open-angle glaucoma), a reduction of the macula pit width and a non-detectable external limiting membrane.
- HH-OCT in children with glaucoma confirmed many similar findings to those in adults (thinned retinal nerve fibre, thinned ganglion cell layer, enlarged cup diameter, smaller rim areas and smaller rim volumes).
- We identify particular threshold parameters to optimise recognition of glaucomatous nerve changes within the studied population.

**How might these results change the focus of research or clinical practice?**

- Sensitivity and specificity of optic nerve changes for a clinical diagnosis of glaucoma suggest that HH-OCT has the potential to be a useful tool to detect disease in young children and is optimised by using the threshold values which this work suggests.
- This is groundwork for a larger prospective study powered to evaluate childhood glaucoma diagnosis and disease control.

## Introduction

Glaucoma is an important cause of preventable visual loss in childhood.^1–3^ Causes for reduced vision include corneal scarring, anisometropic amblyopia, buphthalmia and optic neuropathy.^4 5^ Primary congenital glaucoma (PCG) is one of the most common types of childhood glaucoma comprising 32%^4^–47%^2^ of cases and is usually diagnosed in infancy.^6^ Obstruction of aqueous outflow causes raised intraocular pressure and optic neuropathy.^5^

Assessment of children with glaucoma is challenging because of limited co-operation, media opacity and examination equipment designed for adults rather than paediatric use. While in adults optical coherence tomography (OCT) imaging is standard for assessing glaucoma, infants and young children have not benefited from this technology as they cannot co-operate with standard table-mounted devices. To date, limited literature about the use of OCT in PCG is available.

Retinal nerve fibre layer (RNFL), ganglion cell layer (GCL) thinning and enlarged cup with smaller rim areas/volumes of the optic nerve (ON) were found in a cohort of children of an average age of 10.1±3.6 years after glaucoma surgery on OCT.^7^ Cup:disc ratio (CDR) can be reversible in paediatric glaucoma after surgery. In contrast, RNFL thinning progressed even after treatment and visual prognosis depended mainly on preoperative RNFL thickness.^8^ Retinal changes including cystoid macular oedema, atrophy, pigment epithelial detachment, choroidal folds and/or disruption of the inner/outer photoreceptor segments were found in 13.2% of children with different types of glaucoma over 12 years of age.^9^

The aim of this study was to investigate in PCG (1) the feasibility of scanning the ON and central retina with hand-held OCT (HH-OCT) without sedation or anaesthesia; (2) the characteristics of ON changes in comparison with adult open-angle glaucoma (POAG), and for both groups, comparison with matched controls; (3) the sensitivity and specificity of ON parameters for diagnosis; and (4) changes of the size and shape of the foveal pit and individual retinal layers.

## Methods

In this observational cross-sectional study, we investigated 20 patients with PCG (10 females and 10 males; mean age±SD=4.64±2.79 years), and 20 age-matched, gender-matched and ethnicity-matched healthy controls (9 females and 11 males; mean age 4.73±2.81 years).

All patients were imaged between November 2015 and November 2016. Twenty-four patients were recruited from paediatric glaucoma clinics at Birmingham Children’s Hospital and University Hospitals of Leicester, UK. All eligible sequential patients were included as part of this study. PCG was confirmed based on the presence of clinical features documented in the clinical record (reference test). The ninth Consensus Report of the World Glaucoma Association (http://www.worldglaucoma.org/consensus-9/) criteria were used for PCG diagnosis. Inclusion criteria for the study were two or more of the following (in the absence of other ocular or systemic causes): elevated intraocular pressure (IOP), ON cupping, corneal changes of glaucoma, increasing axial length or myopia. Only clinically confirmed PCG was included. Secondary glaucomas were excluded including all in which structural abnormalities of the anterior segment were found (except isolated goniodysgenesis; a feature of PCG). Those children for whom cooperation or media opacity prevented adequate image acquisition were excluded. Healthy children were recruited from Leicester nurseries and schools and matched by age, gender, ethnicity and clinical/scanning protocol.

To compare ON changes with POAG, we included data from 10 patients with POAG (all patients had visual field defect with ON cupping and RNFL and neuroretinal rim thinning on OCT; https://wga.one/wga/consensus-10/) (two females, eight males, mean age 66.8±6.94 years). POAG cases were recruited from glaucoma clinics at Leicester Royal Infirmary during 12 months from November 2015. POAG controls were recruited from hospital staff and the general public and were matched for age, gender, ethnicity and refractive error.

Ophthalmic examination included best-corrected visual acuity, cycloplegic refraction, slit-lamp and fundus examination and measurement of horizontal corneal diameter and IOP (i-care TAO1i, Finland/Goldman tonometry if possible). Demographic characteristics and clinical data of participants are shown in table 1.

**Table 1:** Clinical and demographic characteristics of children with primary congenital and adults with open-angle glaucoma at inclusion in study

ID	Age (years)	Sex	Ethnicity	Affected eye	Vision (logMAR)		Refraction (SE)		IOP		Corneal diameter (mm)		Cup:disc ratio		Surgical treatment
					RE	LE	RE	LE	RE	LE	RE	LE	RE	LE	
A. Primary congenital glaucoma															
1	10.3	M	A	BE	0.00	−0.06	Plano	Plano	16	18	12	12	0.4	0.3	G(BE)
2	9.3	M	A	BE	1.30	0.08	−9.00	−1.00	20	20	12.5	11	0.4	0.3	G(BE×2)
3	0.8	F	C	BE	0.98 (PL)	FF (PL)	−2.75	−1.75	22	20	14	10	0.3	0.2	G(BE), TRAB(LE)
4	4.3	M	C	BE	0.13	0.13	−0.25	−0.50	15	21	12	12	0.35	0.5	G(BE), TRAB(RE)
5	2.1	F	C	BE	1.10 (CC)	1.10 (CC)	+1.00	Plano	14	16	14	14	0.1	0.1	G(BE), BT(RE)
6	3.7	F	A	BE	0.40 (CC)	0.40 (CC)	+0.50	−0.50	20	20	13	13	0.8	0.8	G(BE)
7	5.8	F	A	BE	0.43	0.70	−6.25	−1.00	14	12	14	13	0.5	0.5	TRAB(BE)
8	8.0	F	A	BE	0.22	0.64	−1.50	1.00	12	12	11	13	0.5	0.5	TRAB(BE×2RE)
9	3.6	F	A	BE	0.20	0.20	+1.75	−2.75	6	11	14	13	0.5	0.5	G(BE)
10	1.5	M	C	RE	0.80 (CC)	0.70 (CC)	−0.25	+2.25	16	14	12	11	0.5	0.5	G(RE×2)
11	0.4	M	C	BE	0.86 (PL)	0.64 (PL)	−1.00	−1.50	29	25	12	13	0.5	0.5	Listed for surgery
12	5.7	M	C	BE	0.15	0.13	−1.00	−1.00	8	8	13	13	0.7	0.4	G(BE)
13	1.6	M	C	LE	0.10 (PL)	0.70 (PL)	−1.00	Plano	14	12	10	13	0.3	0.6	G(LE×2)
14	3.6	M	C	RE	0.95	0.30	−11.00	−0.25	18	8	13	10	0.8	0.3	G(RE)
15	2.9	F	A	LE	0.35	1.06	1.25	−3.50	18	20	10	12	0.2	0.45	G(LE)
16	5.0	F	A	RE	1.83	−0.05	−3.00	+0.75	14	16	12	10	0.2	0.2	G(RE×2), CL(RE)
17	2.3	F	C	BE	0.51	0.51	−1.25	1.50	23	12	23	12	0.2	0.2	G(RE)
18	5.1	M	C	LE	0.08	LP	+0.25	−7.00	28	12	9	12	0.2	0.9	G(LE×3)
19	7.2	M	C	BE	1.15	0.08	+2.25	+2.25	30	18	12	12.5	0.8	0.9	G(BE), TRAB(BE)
20	6.7	F	C	BE	0.10	0.10	−1.00	−1.00	25	23	14	13	0.4	0.9	G(BE)
21	2.1	M	C	BE	0.8	0.7	−0.50	+1.25	14	16	10	12	0.4	0.4	G(BE)
22	0.8	M	A	LE	FF	FF	+1.00	−3.00	12	20	10	12	0.4	0.7	G(LE), TRAB(LE)
23	4.1	F	A	BE	0.4	0.4	Plano	Plano	16	16	12	12	0.5	0.5	G(BE)
24	2.0	M	A	BE	FF	FF	–	–	19	20	12	11	–	–	Listed for surgery
B. Healthy children (controls for primary congenital glaucoma)															
1	0.4	F	C	–	0.4 (PL)	0.4 (PL)	Plano	Plano	12	12	10.5	10.5	0.3	0.3	–
2	0.5	M	C	–	0.3 (PL)	0.3 (PL)	+2.00	+2.00	14	13	11	11	0.2	0.2	–
3	0.7	F	C	–	0.4 (PL)	0.4 (PL)	+3.25	+3.00	11	12	10.8	10.8	0.3	0.3	–
4	0.8	F	C	–	0.3 (PL)	0.3 (PL)	+2.50	+2.50	14	14	11	11	0.3	0.3	–
5	0.8	M	A	–	FF	FF	+2.50	+2.50	12	14	11	11	0.3	0.3	–
6	10	M	A	–	0.3	0.3	Plano	Plano	16	16	11.2	11.2	0.35	0.35	–
7	3	F	C	–	0.1 (CC)	0.1 (CC)	+1.25	+1.25	16	17	11	11	0.2	0.2	–
8	4	F	A	–	0.00	0.00	+1.00	+1.00	16	16	11	11	0.3	0.3	–
9	7	F	A	–	0.10	0.10	Plano	Plano	17	16	11	11	0.3	0.3	–
10	4	M	C	–	0.2 (CC)	0.2 (CC)	+0.75	+0.75	18	18	11	11	0.3	0.3	–
11	6.4	M	C	–	0.15	0.13	Plano	Plano	15	15	10	10	0.3	0.3	–
12	5.5	M	C	–	0.10	0.10	Plano	Plano	16	18	11	11	0.3	0.3	–
13	2	F	A	–	0.3 (CC)	0.3 (CC)	+1.25	+1.25	16	14	11	11	0.3	0.3	–
14	4	M	C	–	0.20	0.20	+1.00	+1.00	16	16	11	11	0.3	0.3	–
15	5	F	A	–	0.10	0.10	+1.25	+1.25	18	18	11	11	0.2	0.2	–
16	5	M	C	–	0.00	0.00	+0.50	+0.75	16	16	11	11	0.2	0.2	–
17	1.5	M	C	–	0.2 (PL)	0.2 (PL)	+0.75	+0.75	18	18	11	11	0.3	0.3	–
18	4	M	A	–	0.10	0.10	+2.00	+2.00	16	16	11	11	0.35	0.35	–
19	4.3	M	A	–	0.00	0.00	+2.00	+2.00	19	20	11	11	0.3	0.3	–
20	3.6	F	A	–	0.10	0.20	+1.50	+1.75	20	20	11	11	0.4	0.4	–
C. Open-angle glaucoma															
1	69	F	C	BE	0.32	0.18	+0.25	+0.5	14	16	12	12	0.8	0.8	TRAB(RE)
2	65	M	C	RE	0.16	0.20	Plano	Plano	20	19	12	12	0.85	0.5	–
3	76	M	C	BE	0.45	0.20	+1	+1	22	22	12	12	0.5	0.7	–
4	76	M	C	BE	0.36	0.2	Plano	Plano	16	15	12	12	0.6	0.5	–
5	59	M	C	BE	0.10	0.10	+0.25	Plano	12	15	12	12	0.7	0.8	–
6	69	F	A	BE	0.10	0.10	Plano	Plano	18	18	12	12	0.8	0.8	–
7	56	M	C	BE	0.14	0.22	Plano	Plano	15	16	12	12	0.8	0.65	TRAB(BE)
8	66	M	C	BE	0.14	0.16	Plano	Plano	13	11	12	12	0.6	0.7	–
9	72	M	C	BE	0.36	0.22	Plano	Plano	14	14	12	12	0.7	0.8	–
10	60	M	C	BE	0.24	0.20	Plano	Plano	16	17	12	12	0.6	0.5	–
D. Healthy adults (controls for open-angle glaucoma)															
1	77	M	C	–	0.10	0.10	Plano	+0.25	19	19	12	12	0.4	0.4	–
2	54	M	C	–	0.20	0.10	Plano	+0.25	17	16	12	12	0.35	0.4	–
3	60	M	C	–	0.10	0.10	Plano	+0.50	14	14	12	12	0.4	0.4	–
4	71	F	A	–	0.20	0.20	+0.75	Plano	12	12	12	12	0.4	0.4	–
5	66	F	C	–	0.20	0.10	+0.50	Plano	16	16	12	12	0.3	0.3	–
6	72	M	C	–	0.10	0.10	Plano	Plano	12	14	12	12	0.5	0.5	–
7	57	M	C	–	0.10	0.20	Plano	Plano	19	20	12	12	0.4	0.4	–
8	71	M	C	–	0.10	0.20	Plano	Plano	18	18	12	12	0.3	0.4	–
9	69	M	C	–	0.20	0.10	Plano	Plano	16	16	12	12	0.4	0.4	–
10	72	M	C	–	0.10	0.10	Plano	Plano	20	20	12	12	0.5	0.45	–

IOP was measured at the date when optical coherence tomography was done.

BE, both eyes; BT, Baelvedt tube; C, Caucasian; CC, Cardiff cards (in all other participants, Kay pictures or Snellen test was used); CL, cyclodiode laser; F, female; FF, following and fixing; G, goniotomy; IOP, intraocular pressure; LE, left eye; M, male; PL, perception of light; PL, preferential looking; RE, right eye; SE, spherical equivalent; TRAB, trabeculectomy.

### OCT imaging

HH-OCT was performed without anaesthesia or sedation in outpatient clinics by research orthoptists without knowledge of the specific clinical details and diagnosis of the patients. A hand-held SD-OCT device (Envisu 2300; Leica Microsystems, Germany) 840 nm wavelength, 2.6 mm theoretic axial resolution was used. ON and macula imaging was carried out using 12 mm wide by 8 mm high scans with 80 horizontal B-scans composed of 600 A-scans and a 1.67 s acquisition time. To ensure central ON and fovea position of the B-scan, position was verified on the en-face view.

**Figure 1:**
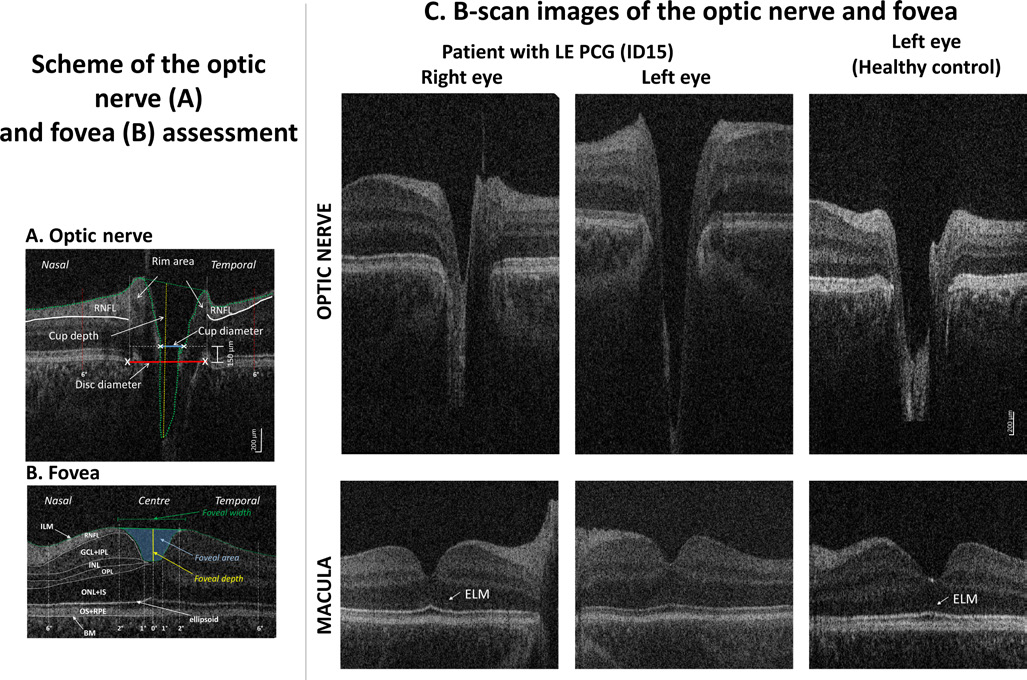
Hand-held optical coherence tomography (HH-OCT) images of the optic nerve (ON) (A) and fovea (B) of the left eye of patient 6: (A) horizontal B-scan through the centre (deepest excavation) of the optic disc; the disc diameter was defined as an interval between the edges of Bruch’s membrane (red line), the cup diameter as the distance between the nasal and temporal internal limiting membrane (green dotted line) 150 µm anterior to the plane of the disc (blue line), the rim area consisted of the area anterior to the same plane (white dotted lines) within the disc edges (white vertical lines) and the internal limiting membrane (green dotted lines); maximal cup depth (vertical yellow line) was measured using a line perpendicular to the line between the cup diameter (blue line) and the deepest point of the cup; RNFL thickness was measured at 6° from disc margins (red dotted lines). (B) Horizontal B-scan of the fovea with labelled individual retinal layers (BM, Bruch’s membrane; ILM, inner limiting membrane; RNFL, retinal nerve fibre layer; GCL, ganglion cell layer; INL, inner nuclear layer; IPL, inner plexiform layer; IS, inner segment; ONL, outer nuclear layer; ELM, external limiting membrane; OPL, outer plexiform layer; OS, outer segment; RPE, retinal pigment epithelium). Retinal layer thickness was measured in the centre of the fovea, in the paracentral area (from 1° nasally to 1° temporally) and nasally and temporally (from 2° to 6°). The green line connecting the most prominent positions of the ILM nasally and temporally was used to define the foveal width; the yellow line indicates the foveal depth (the axial distance from the green line to the deepest point of the foveal pit); the area in blue indicates the foveal pit area. (C) Horizontal spectral domain–optical coherence tomography B-scan images of the optic nerve (top) and fovea (bottom) of patient 20 with primary congenital glaucoma in the left eye (PCG, middle column), an unaffected left eye (left column) and an eye of a healthy age-matched, gender-matched and ethnicity-matched control child (right column). On the ON scan, a larger and deeper cup is seen in PCG. On the foveal scan, the ELM is not visible in PCG while it is distinctly seen in the unaffected eye and in the healthy control.

Masked optic nerve head analysis was performed on one B-scan through the centre of the ON cup using a custom-written ImageJ macro (National Institutes of Health, Bethesda, Maryland, USA; available at http://rsbweb.nih.gov/ij/; accessed 3 March 2016) by SS. Cup and disc measurements are derived as shown in figure 1A from a plane of 150 µm anterior to the disc edges. Parameters included horizontal disk and cup diameter, CDR, cup area, rim width (temporal/nasal width combined), rim area (temporal/nasal combined), and nasal and temporal RNFL. Cup depth was calculated as the axial distance from the cup bottom to a line connecting the nasal and temporal rim edges (figure 1A).

Lateral measures were converted to visual angle using a conversion factor provided by Leica Microsystems derived using a model eye (292 µm=1° angle for small angles). This addresses the problems of calibration errors of lateral distance measures caused by changing axial lengths in the developing eye and in eyes with severe refractive error present in children with glaucoma.

Segmentation of the foveal region masked to PCG or control was performed using ImageJ and included the RNFL, and inner nuclear (INL) and outer plexiform (OPL) layers.^10^ The outer nuclear layer (ONL) and inner segment (IS), the outer segment and retinal pigment epithelium layers, the GCL and inner plexiform (IPL=ganglion cell complex) layers were combined for analysis due to poor contrast difference.^11 12^ Layer thicknesses were averaged in the paracentral area (from 1° nasally to 1° temporally from the foveal pit bottom) as well as in the nasal and temporal areas (from 2° to 6° from the foveal pit bottom). The inner limiting membrane on the flattened B-scan image was used to calculate foveal pit depth, width and area (figure 1B).^10^

### Statistical analysis

SPSS software V.16.0 was used. ON head and macular parameters were normally distributed. Separate linear mixed models were generated for each of the OCT parameters to allow inclusion of repeated measures data (ie, eye) and non-repeated measures data (ie, group and age) in the same statistical models. In each model, the OCT parameter was the outcome variable, participant-anonymised ID was included as a random factor; group and eye were included as fixed factors; and age as a covariate. Bonferroni correction was used to correct for the multiple comparisons. To compare glaucoma between children and adults, each ON parameter was expressed as percentage of the mean control value.

Sensitivity and specificity of ON parameters for PCG detection were calculated using fixed thresholds for each ON parameter that demonstrated statistically significant difference.

Correlations between ON and macular parameters, IOP and horizontal corneal diameter were calculated using Pearson’s correlation. P value ≤0.05 was considered statistically significant.

## Results

### ON head morphology

Four patients were excluded from analysis; in two children, the affected eye could not be assessed due to low scan quality secondary to corneal oedema, and two children did not co-operate (table 1, patients 21–24). Data from both eyes of 20 out of 24 patients with PCG were included (83.3% of eyes).

Mean IOP before OCT examination was 17.68±6.52 in PCG and 22.12±4.16 in POAG.

Visual inspection showed marked cupping with enlarged horizontal cup diameter and deeper cup depth in most patients (figure 1C).

In PCG, ON analysis showed significantly increased CDR, cup diameter, cup area and cup disc. Rim width, rim area and nasal RNFL were significantly reduced in PCG. In contrast, in adult glaucoma, we did not find significant differences in cup depth and nasal RNFL as compared with their controls.

**Figure 2:**
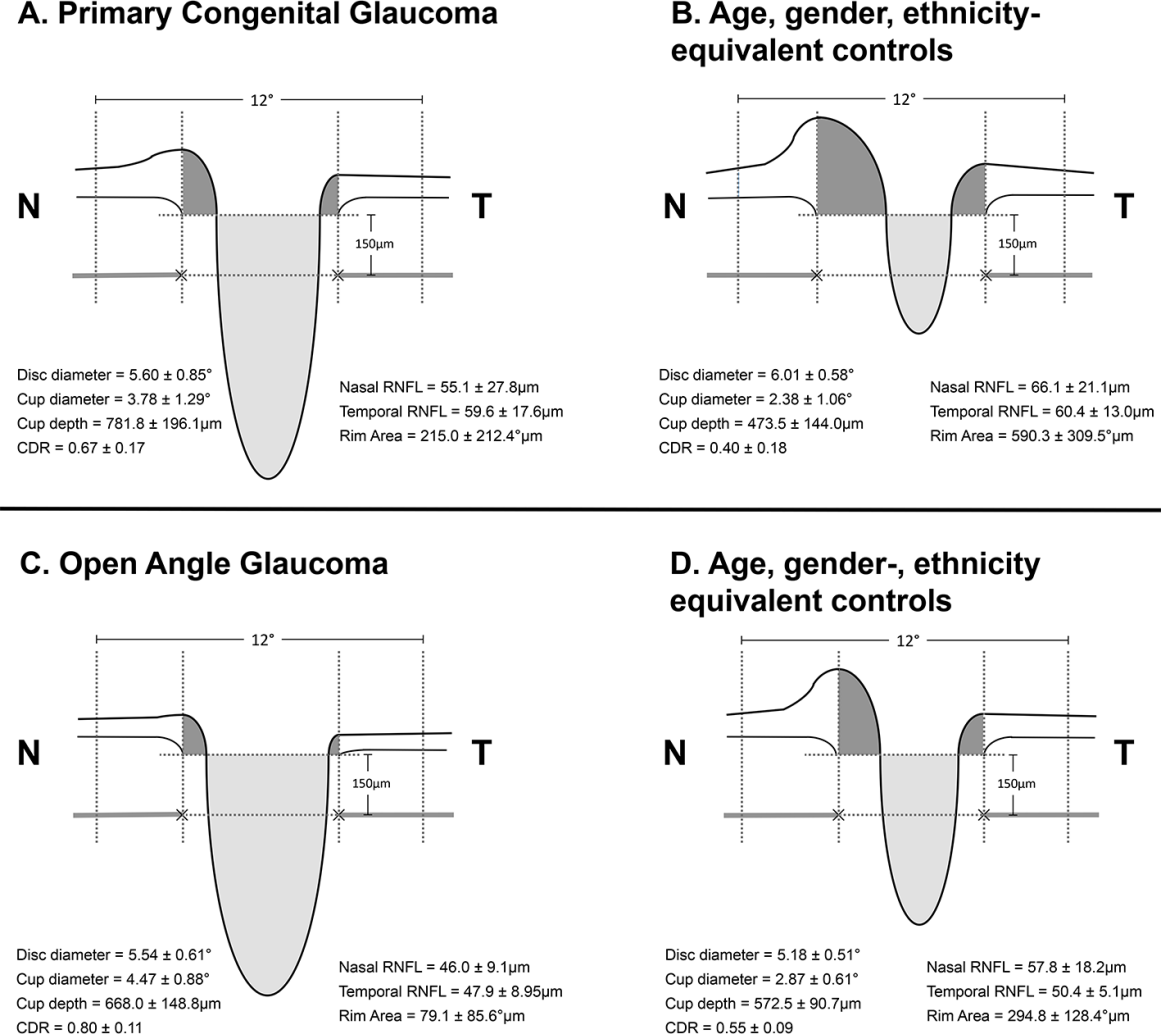
Cross-sectional schematic diagrams representing mean values of optic nerve head parameters of patients with primary congenital glaucoma, adult open-angle glaucoma and matched healthy controls for each group (numeric values represent mean±SE). The upper horizontal dotted lines represent the horizontal offset (150 µm) used to determine cup diameters and the lower horizontal dotted lines indicate disc horizontal diameters. The vertical dotted lines show the margins of the rim areas. CDR, cup:disc ratio; N, nasal; RNFL, retinal nerve fibre layer; T, temporal.

Children with PCG had significantly deeper cup depth (165% increased from control children) than patients with POAG (117% from adult controls) (p=0.014) (table 2C and figure 2).

**Table 2:** (A) Mean (±SD) of OCT optic nerve parameters in children with PCG and age-equivalent, gender-equivalent and ethnicity-equivalent healthy controls (the two groups were compared statistically using linear mixed models); (B) mean (±SD) of optic nerve parameters in adults with POAG and age-equivalent, gender-equivalent and ethnicity-equivalent healthy controls (the two groups were compared statistically using linear mixed models); (C) comparison of optic nerve parameters relative to their control values (patient values/mean control value×100%) in children with PCG and adults with POAG

(A) Children with PCG compared with controls				
	PCG	Controls	Linear mixed model	
	Mean±SD	Mean±SD	F	P value
Disc diameter (°)	5.596±0.851	6.014±0.578	3.28	0.078
Cup:disc ratio	0.668±0.173	0.398±0.178	26.46	**<0.000**
Cup diameter (°)	3.782±1.288	2.381±1.061	15.65	**<0.000**
Cup area (µm)	1491.3±778.2	503.6±451.7	25.93	**<0.000**
Cup depth (µm, from rim edges)	781.8±196.1	473.5±144.0	34.08	**<0.000**
Rim width (°)	1.814±0.947	3.633±1.198	31.87	**<0.000**
Rim area (µm)	215.0±212.4	590.3±309.5	21.62	**<0.000**
Nasal RNFL at 6° from disc centre (µm)	51.1±20.0	66.1±21.1	5.29	**0.028**
Temporal RNFL at 6° from disc centre (µm)	59.6±17.6	60.4±13.0	0.02	0.894
Age (years)	4.45±2.82	4.73±2.81	0.14	0.711
**(B) Adults with POAG compared with controls**				
	**Adult glaucoma**	**Controls**	**T-test**	
	**Mean ± SD**	**Mean ± SD**	**F**	**P value**
Disc diameter (°)	5.536±0.612	5.180±0.513	1.47	0.175
Cup:disc ratio	0.804±0.109	0.551±0.087	8.17	**0.000**
Cup diameter (°)	4.470±0.876	2.866±0.612	6.18	**0.000**
Cup area (µm)	1636±633.4	695±175.6	5.79	**0.000**
Cup depth (µm, from rim edges)	668.0±148.8	572.5±90.7	1.83	0.100
Rim width (°)	1.066±0.531	2.314±0.449	8.00	**0.000**
Rim area (µm)	79.03±85.6	294.8±128.4	5.61	**0.000**
Nasal RNFL at 6° from disc centre (µm)	46.0±9.08	57.8±18.19	1.86	0.096
Temporal RNFL at 6° from disc centre (µm)	47.9±8.95	50.4±5.1	0.74	0.479
Age (years)	67.0±6.96	66.8±7.50	0.07	0.949
**(C) Comparison of optic nerve parameters relative to their control values**				
	**PCG**	**Adults**	**Linear mixed model**	
	**Mean ± SD**	**Mean ± SD**	**F**	**P value**
Disc diameter (°)	93%±14%	107%±12%	8.64	**0.006**
Cup:disc ratio	168%±43%	146%±20%	1.83	0.186
Cup diameter (°)	159%±54%	156%±31%	0.00	0.966
Cup area (µm)	296%±155%	235%±91%	0.26	0.611
Cup depth (µm, from rim edges)	165%±41%	117%±26%	6.65	**0.014**
Rim width (°)	50%±26%	46%±23%	0.14	0.708
Rim area (µm)	36%±36%	27%±29%	0.95	0.337
Nasal RNFL at 6° from disc centre (µm)	83%±42%	76%±32%	0.39	0.538
Temporal RNFL at 6° from disc centre (µm)	99%±29%	97%±17%	1.01	0.323

OCT, optical coherence tomography; PCG, primary congenital glaucoma; POAG, primary open-angle glaucoma; RNFL, retinal nerve fibre layer.

Differences in disc diameters were not significant for PCG and adult glaucoma (vs age-matched controls, table 2A and B); however, since changes deviated in opposite directions, the relative differences were significant, with PCG apparently having smaller disc diameters than controls and adults with POAG having larger disc diameters (table 2C).

**Figure 3:**
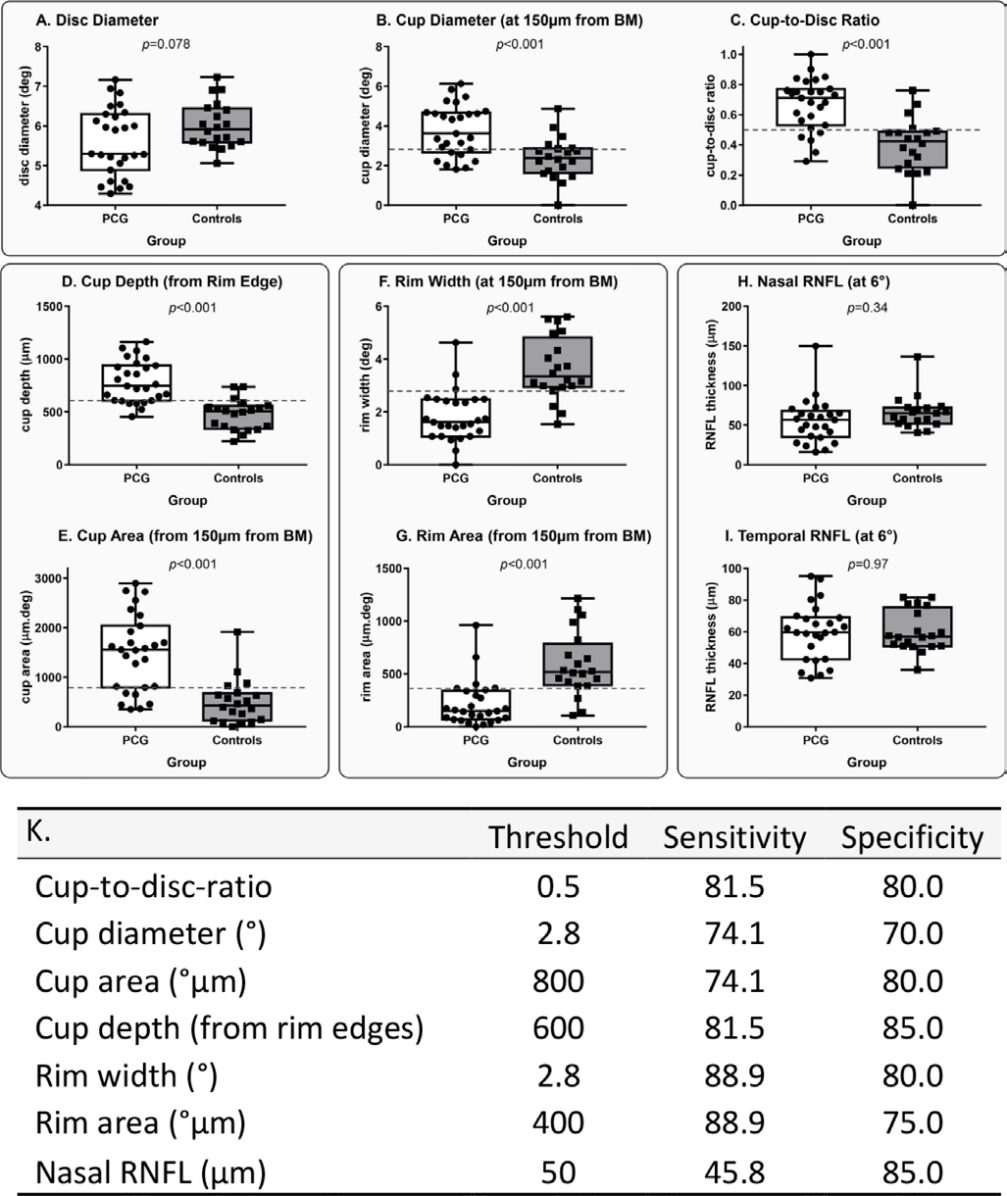
(A–I) Distribution of optic nerve head parameters in patients with primary congenital glaucoma (PCG) and healthy controls. Horizontal dotted lines show optimal sensitivity and specificity thresholds for the optic nerve parameters that were highly sensitive and specific for PCG detection. BM, Bruch’s membrane; RNFL, retinal nerve fibre layer. (K) Optimal sensitivity and specificity thresholds, sensitivity and specificity analysis for optic nerve head parameters in patients with primary congenital glaucoma compared with healthy controls.

Optimum threshold ON HH-OCT parameters were used to generate sensitivity, specificity, positive predictive values (PPVs) and negative predictive values (NPVs) for OCT detection of clinically diagnosed PCG (figure 3). Sensitivity values for the five ON HH-OCT morphological categories CDR, cup diameter, cup depth, and rim width and rim area were respectively 81.5, 74.1, 81.5, 88.9% and 88.9%. Respective specificity values were 80.0, 70.0, 85.0, 80.0% and 75.0% (figure 3). These are presented in table 3 along with 95% CIs.

**Table 3:** Optic nerve threshold values which optimise sensitivities, specificities, and positive and negative predictive values (with 95% CIs)

	Threshold	Sensitivity (%)			Specificity (%)			Positive predictive value (%)			Negative predictive value (%)		
		Value	LCI	UCI	Value	LCI	UCI	Value	LCI	UCI	Value	LCI	UCI
Cup:disc ratio	0.5	81.5	61.2	93.7	80.0	56.3	94.3	84.6	69.2	93.1	76.19	58.5	87.9
Cup diameter (°)	2.8	74.1	53.7	88.9	70.0	45.7	88.1	76.9	62.2	87.1	66.7	49.9	80.1
Cup depth (from rim edges)	600	81.5	61.9	93.7	85.0	62.1	96.8	88.0	71.8	95.5	77.3	60.1	88.5
Rim width (°)	2.8	88.9	70.8	97.7	80.0	56.3	94.3	85.7	71.2	93.6	84.2	64.2	94.1
Rim area (µm)	400	88.9	70.8	97.7	75.0	50.9	91.3	82.8	69.0	91.2	83.3	62.6	93.7

LCI, lower CI;UCI, upper CI.

### Foveal morphology

On visual inspection (figure 1C bottom), the ELM was not visible in any of the patients with PCG and did not allow distinguishing between ONL and IS.

Analysis of individual retinal layers of the central horizontal B-scan did not reveal any significant changes except the thickening in the INL pericentrally in the temporal retina in the PCG group as compared with controls (F=5.55; p=0.024).

The foveal width in the PCG group was smaller (87%; 8.38±1.00 and 9.62±0.65, p<0.001) than in controls.

## Discussion

This study represents the largest cohort of atients with PCG that have been imaged using HH-OCT to date. OCT was successful in 83.3% eyes of the patients with PCG without sedation or anaesthesia.

In our study, children with PCG had significantly deeper cups than patients with POAG underlining the importance of measuring cup depth in addition to CDR. Changes of collagen fibril diameter and increased elasticity of the lamina cribosa early in life have been found in animal experiments.^13^ Possibly increased elasticity of the lamina cribrosa in childhood leads to increased deepening of the cup in PCG. Differences in collagen elasticity have also been postulated to explain reversing of cupping after surgical reduction of IOP in PCG.^14^ We did not find correlation of the IOP on the day of OCT acquisition with cup depth or other OCT parameters; however, this study did not include serial measurements of particular individuals which we, like others,^14^ have found to show reversibility of cupping on OCT, and most of our patients with PCG had controlled IOP at the time of imaging (mean IOP was 17.68 mm Hg).

Standard ON assessment of adult patients with glaucoma includes the measurement of the average RNFL thickness of the peripapillary area on OCT. In our study, we have found significantly thinner RNFL in PCG in the nasal segment only using one single horizontal B-scan. It is likely that volumetric peripapillary RNFL thickness analysis involving all quadrants around the ON is more sensitive for assessment of ON damage in PCG and POAG. RNFL thinning starts with the inferior and superior segments and then involves nasal and temporal parts in adults.^15 16^ Therefore, nasal and temporal segments might be suboptimal to reflect glaucomatous changes. Similarly, assessment of vertical cup diameter might show earlier changes in PCG. However, compliance with acquiring vertical scans is more difficult due to the necessity of wide opening of the upper lid. In a few studies investigating RNFL thinning in patients with juvenile glaucoma, only the difference in superior and inferior segments was statistically significant compared with controls.^17 18^ Due to fixation instability of young children and infants, it is difficult to obtain volumetric OCT scans covering all quadrants around the ON. A refined faster acquisition protocol with fewer B-scans has good potential to improve compliance with vertical and volumetric scans and to further increase specificity and sensitivity of OCT to detect PCG (unpublished data).

CDR measured by OCT in adult patients with glaucoma was found to be larger as compared with clinical assessment.^19^ CDR was significantly larger on OCT than judged on funduscopy in our patients with PCG. This could be explained by differences in assessment such as judgement of an area on clinical examination compared with measurement of a single horizontal B-scan. Other possibilities to explain the smaller CDR on clinical examination are obscuration of the cup by vessels and/or assessment of the cup size at a deeper level on funduscopy than by using OCT where a default setting of 150 µm above the Bruch’s membrane was used. It is likely that ON parameters obtained using OCT are more reliable, at least longitudinally, than clinical judgement using ophthalmoscopy. However, as with RNFL, it would be preferable for the CDR to be assessed for the entire nerve.

Cup depth, cup diameter, CDR and rim width/area show useful sensitivity (74.1%–88.9%) and specificity (70.0%–85.0%) parameters for PCG detection. The thresholds of 600 µm, 2.5°, 0.5 and 2.8/400 µm, respectively, found in our study could be selected for screening children suspected of glaucoma with OCT. Cup depth was the most specific parameter (85%). Moreover, it is one of the easiest parameters to analyse as it can be measured using callipers available on the HH-OCT machine without the need of segmentation.

It is important to recognise the limitations of our analysis of HH-OCT as a diagnostic tool. We used HH-OCT to identify childhood glaucoma in a specific clinical cohort with disease which differs from non-selected screening; this cohort has moderate to severe surgical PCG and differs from a non-targeted screening population in prevalence and severity of disease. The parameters we describe (sensitivity, specificity, PPV, NPV) apply to this cohort. We chose to study PCG (vs controls) because here, early-onset disease allowed us to characterise ON morphology at younger ages and the aggressive nature of the disease gives a higher prevalence of those structural changes. This allows the study to establish feasibility and characterise the differences between the groups. The diagnostic accuracy of HH-OCT or any diagnostic test is contextual,^20^ and accuracy parameters (and optimised threshold values) would differ in studies of different cohorts. We advocate studies, where the objective, using STARD^20^ methodologies, is to establish whether earlier diagnosis with HH-OCT would ultimately improve outcome.

The clinical presentation of PCG differs from POAG in that the disease is usually symptomatic and the diagnosis is more often obvious to practitioners familiar with the condition, so the need for a tool for screening is less. We foresee that the primary use of HH-OCT in PCG is for disease monitoring. This study into the diagnostic use of HH-OCT informs which ON head parameters are most likely to yield useful disease monitoring information in these children and to prioritise those parameters in imaging algorithms: cup depth is promising with highest specificity and PPV of the ON parameters studied (85% and 88%, respectively). We would advocate a longitudinal cohort study to evaluate HH-OCT disease monitoring of PCG and suggest that those ON parameters presented here might inform such work. The use of HH-OCT as a *diagnostic* tool in childhood glaucomas will likely be greatest in non-PCG glaucomas which can present relatively insidiously, such as distinguishing GCFCs (glaucoma following cataract surgery/aphakic glaucoma) from ocular hypertension in this group.

In a recent study, outer retinal layer changes and isolated INL cysts were found in a heterogeneous group of patients with paediatric glaucoma as well as non-glaucomatous optic atrophy.^9^ We have not observed cysts or changes in the outer and inner retinal layers except the absence of the ELM in all patients with PCG. Thickening of the INL in our study confirmed the results of Silverstein *et al*.^21^ However, missing ELM have not been described previously and could be caused by pathological retinal development and/or photoreceptor damage due to the raised IOP in PCG. ELM loss has been proposed to be a feature of early photoreceptor damage and associated with reduced blood supply to the retina.^22 23^ It is possible that the ELM, which is a structure with high oxygen demand, is more vulnerable at a young age when retinal development is not complete. Therefore, hypoxia induced by increased IOP could trigger retinal damage in PCG. Another possibility causing ELM changes could be reduced scan quality due to signal loss with corneal oedema/scarring.

We found significant reduction of the foveal pit width. It is reported in the literature that patients with POAG have progressive retinal thinning which is related to GCL loss.^24 25^ We have not found significant GCL thinning; however, statistically significant pit changes could be an early sign of GCL loss on OCT. The fovea is developing dramatically in the first years of life with inner retinal layers moving away from the fovea.^12^ Possibly the increase in pressure early in life changes the dynamics of foveal development.

The number of patients was relatively small and ON and foveal analyses were based only on single horizontal B-scan images rather than volumetric analysis to allow fast scans in children with poor co-operation. Consequently, the described changes do not represent all the structural abnormalities in patients with PCG that could be captured by volumetric analysis. Vertical scans are probably more likely to detect early changes, as is seen in POAG.

In conclusion, our study shows that HH-OCT in children with PCG is feasible in a large percentage of children without sedation or anaesthesia. ON changes in children showed increased CDR, similar to POAG; however, excavation of the cup was deeper in children.

HH-OCT has the potential to be a useful tool for detecting glaucoma in young children. These findings form the groundwork for an expanded prospective study powered to evaluate HH-OCT for both disease monitoring in PCG and disease detection in non-PCG cohorts.
